# IRN2Vec: A representation learning model for road network intersections by integrating geospatial attributes and travel behaviors

**DOI:** 10.1371/journal.pone.0344448

**Published:** 2026-03-13

**Authors:** Xiaobo Yang

**Affiliations:** Department of Information Science and Technology, Zhejiang Shuren University, Hangzhou, Zhejiang Province, P. R.China; National University of Defense Technology, CHINA

## Abstract

The structural characterization of road networks serves as a critical foundation for enabling high performance in intelligent transportation systems. This paper proposes IRN2Vec, an intersection-oriented representation learning model that generates discriminative road intersection embeddings by integrating geospatial attributes, semantic homogeneity, and mobility behavior features through the LEIRN framework. The model employs a shortest-path sampling strategy to construct training data and adopts a multi-task learning approach to jointly optimize three types of relationships: geographical proximity, label consistency, and categorical similarity. Experiments conducted on real-world road network data from San Francisco, Porto, and Tokyo demonstrate that IRN2Vec achieves average improvements in F1-Score of 31.6%/25.1%, 16.2%/8.6%, and 27.8%/20.2% over UID, GCN, and GAT models, respectively, in traffic signal classification and pedestrian crossing classification tasks. In travel time estimation, it reduces the mean absolute error (MAE) by 12.2%–24.6%. The findings provide effective feature support for traffic state perception and road network optimization.

## Introduction

In urban computing research, traffic data serves as the core foundation for addressing numerous urban issues, making in-depth analysis of intelligent transportation spatiotemporal data critically significant. As the most fundamental and vital component of multi-source heterogeneous traffic data, the structural representation of road networks directly influences the effective performance of system functionalities. Among these, the digital representation of intersections is particularly crucial, forming the foundational support for various intelligent transportation systems (ITS) to achieve their functions [1]. To enhance the efficacy of ITS applications, constructing intersections and road segments with high-quality features has become an urgent requirement in modern road infrastructure development [2–5]. Such features can effectively capture the intrinsic complexities of road networks, including their topological connectivity, spatial distribution patterns, and the homogeneous characteristics existing among different intersections.

In practical applications of Intelligent Transportation Systems (ITS), the performance of downstream tasks such as traffic prediction, route planning, and infrastructure management hinges on the quality of the learned representations of fundamental network elements—namely, intersections. An ideal representation must holistically encode multiple facets: the topological connectivity (how intersections are linked), spatial-geographic attributes (their physical location and proximity), and semantic homogeneity (shared functional or categorical properties, e.g., both being signalized intersections). Relying on manually engineered features for these facets is inefficient and often incomplete.

Recent advancements in Representation Learning, particularly in self-supervised and unsupervised learning, offer a powerful alternative by automatically extracting useful implicit feature vectors (embeddings) from data [6–8]. This paradigm, central to modern AutoMLframeworks, aims to reduce reliance on manual feature engineering—which is often incomplete, biased, and non-scalable—by allowing models to discover discriminative patterns directly from the data structure itself. Our work follows this principle: the ‘implicit feature vectors’ for intersections are not hand-crafted but are learned by the model to simultaneously satisfy multiple relational constraints derived from the raw network and trajectory data. While highly successful in domains like natural language processing and social network analysis [9–11], its direct application to road networks faces distinct challenges, which define the motivation and novelty of our work: 1) Limitation in Incorporating Rich Attributes: Standard network embedding methods like GCN and GAT are designed primarily to capture topological structure. They lack a native mechanism to integrate crucial multi-modal side-information inherent to road networks, such as geographic coordinates, intersection type, or traffic control labels. This results in representations that are structurally aware but semantically and geographically impoverished. 2) Limitation in Behavioral Realism of Sampling: These methods typically rely on random walk strategies for network exploration. However, movement on a road network is not random; users follow purposeful, often shortest or fastest paths between origins and destinations. Random walks fail to capture this fundamental travel behavior, leading to sampled node contexts that may not reflect realistic traffic flow or functional relationships. This hybrid approach, which integrates disparate data modalities (topological, spatial, semantic) into a unified learning model, aligns with the demonstrated effectiveness of hybrid deep learning methods in managing the complexity and high volume of data in IoT ecosystems.

To overcome these limitations, this paper proposes IRN2Vec (Intersection of Road Network to Vector), a novel representation learning model that generates discriminative intersection embeddings. The core innovations and contributions of this work are: 1) A Multi-Task Learning Framework: We propose a unified neural model that jointly optimizes for three key relationships between intersections: (a) Geographical proximity, ensuring spatial locality is preserved; (b) Label consistency, enforcing similarity between intersections with identical traffic signs or rules; and (c) Type similarity, grouping intersections of the same functional category. This framework directly addresses the first limitation by seamlessly integrating topological, spatial, and semantic attributes into a single embedding. 2) A Behaviorally-Grounded Sampling Strategy: Moving beyond random walks, we introduce a shortest-path sampling strategy to construct the training corpus. By simulating realistic user trips, this strategy samples node sequences that align with actual travel patterns, thereby capturing more meaningful topological and functional contexts. This directly tackles the second limitation. 3) The LEIRN PipeGAT: We implement these ideas within a practical two-stage framework (LEIRN) for training and evaluation on large-scale, real-world data. 4) Comprehensive Empirical Validation: Through extensive experiments on road networks from three major cities, we demonstrate that IRN2Vec significantly outperforms state-of-the-art baseGATs (UID, GCN, GAT) in tasks including traffic signal classification, pedestrian crossing classification, and travel time estimation. An ablation study further confirms the individual and complementary value of our proposed sampling strategy and multi-task learning objectives.

These representations are typically learned from data generated by a pervasive Internet of Things (IoT) ecosystem, which includes sensors (e.g., loop detectors, traffic cameras, GPS units), communication networks, and data platforms that collectively enable the collection and transmission of the spatiotemporal traffic data used in our model.

By integrating geospatial attributes, semantic homogeneity, and realistic travel behavior patterns into network topology learning, the IRN2Vec model provides a superior foundational representation for a wide range of ITS applications.

### Developing the IRN2Vec representation learning model

**Fig 1:**
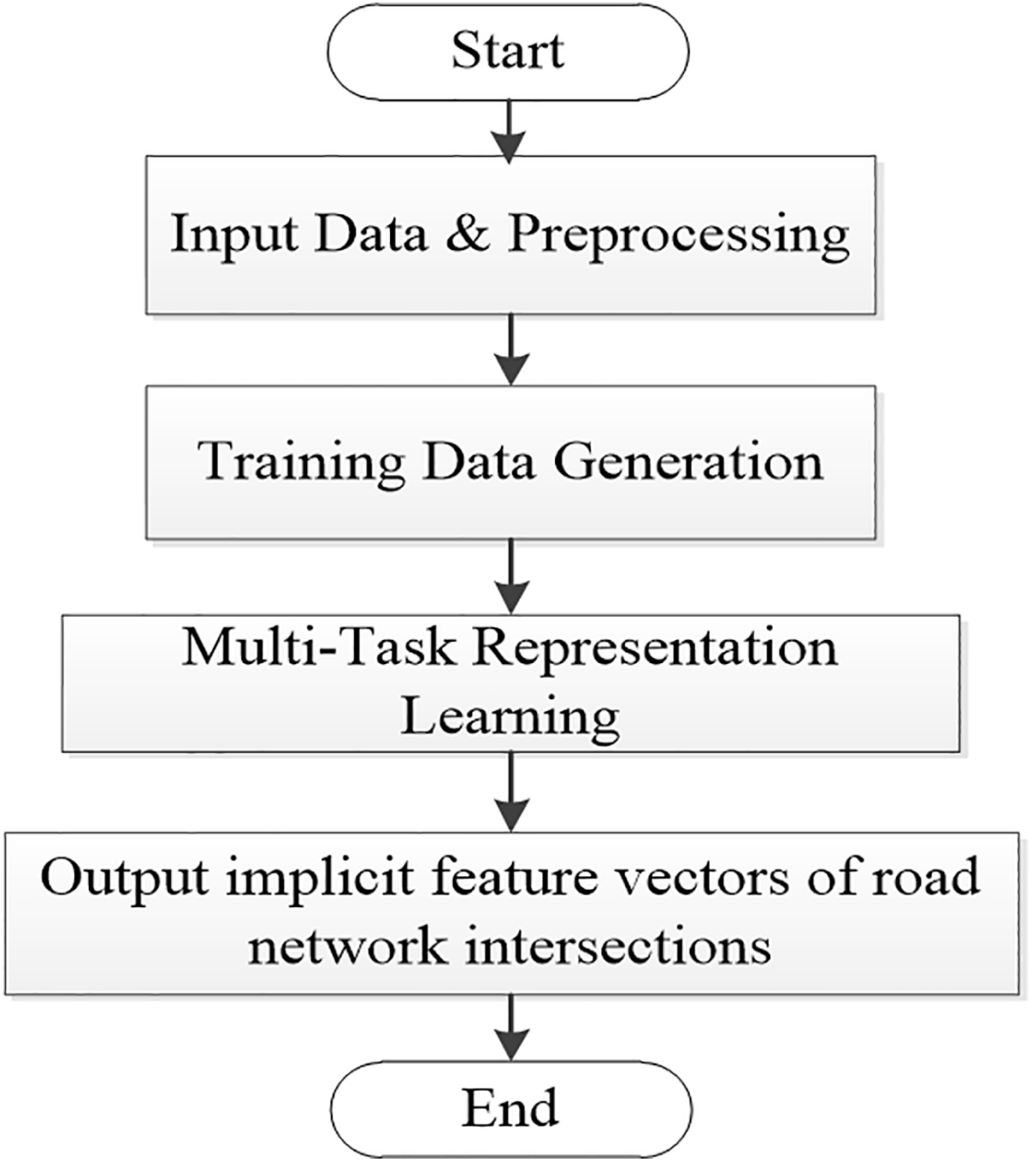
LEIRN overall structural framework.

To construct the IRN2Vec model, this paper designs an overall architecture named LEIRN (Learning Embeddings for Intersection Road Networks) for training and learning implicit feature vectors of road network intersections. As illustrated in (Fig 1). The LEIRN framework is designed to translate raw, heterogeneous transportation data into informative intersection embeddings through two novel, integrated stages.

Stage 1: Behavior-Aware Training Data Generation. This stage moves beyond the classical random walk paradigm. It begins with a road network graph and trajectory data [12]. After map-matching trajectories to the network, our core shortest-path sampler randomly selects origin-destination (OD) pairs and computes the connecting shortest path using the Dijkstra algorithm, simulating realistic user trips. For each pair of intersections (v_i, v_j)co-occurring within a sampled path, we generate a training instance. Crucially, each instance is annotated with three relationship flags based on real-world attributes: a geographic proximity flag (based on Euclidean distance K), a label consistency flag, and a type similarity flag. This process yields a rich corpus of labeled node-pair samples. OD pairs for shortest-path computation are selected via uniform random sampling from the set of all intersection nodes. This ensures comprehensive network coverage and avoids bias towards heavily trafficked routes observed in the trajectory data. For each epoch, we sample a fixed number of OD pairs (e.g., 100,000 per city) to maintain a consistent and manageable training corpus size.

Stage 2: Multi-Task Intersection Representation Learning. The generated triples are used to train the IRN2Vec model. The model learns a single, shared embedding matrix R where each row corresponds to an intersection’s latent vector [13]. For an input pair (v_x, v_j), their embeddings r_x and r_y are retrieved from R. These embeddings are then evaluated simultaneously by three distinct objective functions (corresponding to the three relationship types) within a multi-task learning setup. The model parameters are optimized so that the embeddings accurately predict these relationships, thereby fusing topological, spatial, and semantic information into the final vector representations R.

**Fig 2:**
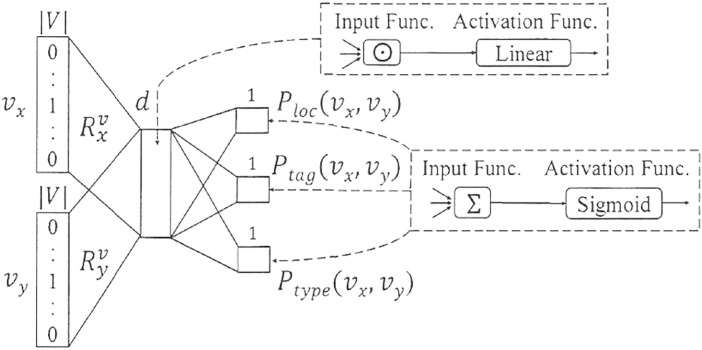
Architecture of the IRN2Vec Model.

The learning of implicit feature vectors for road network intersections aims to extract the common intrinsic attribute characteristics among different intersections, such as whether they share the same functional category or structural type. In the framework of this paper, if two nodes possess certain common attributes, they are considered to form a specific relationship. Additionally, the geo-spatial locality between intersections is also regarded as a type of relationship; for instance, when the spatial distance between two intersections falls within a specific threshold, an association is established. Based on this, a neural network-based representation learning model named IRN2Vec is designed in this paper, which can achieve efficient and rich vector representations of road network intersections by jointly predicting the relationships between any pair of nodes. The structure of the IRN2Vec model is illustrated in (Fig 2).

According to Fig 2, the IRN2Vec model employs a multi-task learning framework to implement a binary relationship classification method based on node pairs. The model takes two nodes, ${\text{v}}_{\text{x}}$ and ${\text{v}}_{\text{y}}$, as input and simultaneously determines whether they satisfy three specific relationships: geographical proximity, intersection label consistency, and intersection type similarity. In the IRN2Vec model, the input to the model is a node pair ${\text{v}}_{\text{x}},{\text{v}}_{\text{y}}\subseteq \text{V}$, with V denoting the node set comprising elements like road network intersections. The core of the IRN2Vec model is a shared embedding lookup table R ∈ ℝ^{|V| × d}, where |V| is the total number of intersection nodes and d is the predefined latent dimension. For an input node pair (v_x, v_y), the model retrieves their corresponding dense vector representations: r_x = R[v_x] and r_y = R[v_y], where r_x, r_y ∈ ℝ^d. These vectors r_xand r_y are the latent embeddings to be learned. The relationship prediction for any task is then computed as the sigmoid of the dot product between these two vectors, e.g., P_loc(v_x, v_y) = σ(r_x· r_y). Therefore, R is the model’s fundamental parameter matrix, and each row R[i] is the final d-dimensional feature vector (embedding) for intersection node i after training. Subsequently, the model uses the vector inner product operation along with a GATar function to predict whether a specific relationship exists between the two road network intersection nodes ${\text{v}}_{\text{x}}$ and ${\text{v}}_{\text{y}}$. Finally, at the output layer, the summation is used as the input function, and the Sigmoid function is activated to compute $\text{Sigmoid}\left({\text{R}}_{\text{x}}^{\prime }{\text{v}}_{\text{x}}\cdot {\text{R}}_{\text{y}}^{\prime }{\text{v}}_{\text{y}}\right)$ for predicting the three previously mentioned specific relationships.

The joint probabilities for three specific relationships—${\text{P}}_{\text{loc}}({\text{v}}_{\text{x}},{\text{v}}_{\text{y}})$、${\text{P}}_{\text{tag}}({\text{v}}_{\text{x}},{\text{v}}_{\text{y}})$、${\text{P}}_{\text{type}}({\text{v}}_{\text{x}},{\text{v}}_{\text{y}})$—are defined as follows:


$${\text{P}}_{\text{loc}}({\text{v}}_{\text{x}},{\text{v}}_{\text{y}})=\sigma (\sum {\text{R}}_{\text{x}}^{\prime }{\text{v}}_{\text{x}}\cdot {\text{R}}_{\text{y}}^{\prime }{\text{v}}_{\text{y}})$$ (1)

$${\text{P}}_{\text{tag}}({\text{v}}_{\text{x}},{\text{v}}_{\text{y}})=\sigma (\sum {\text{R}}_{\text{x}}^{\prime }{\text{v}}_{\text{x}}\cdot {\text{R}}_{\text{y}}^{\prime }{\text{v}}_{\text{y}})$$ (2)

$${\text{P}}_{\text{type}}({\text{v}}_{\text{x}},{\text{v}}_{\text{y}})=\sigma (\sum {\text{R}}_{\text{x}}^{\prime }{\text{v}}_{\text{x}}\cdot {\text{R}}_{\text{y}}^{\prime }{\text{v}}_{\text{y}})$$ (3)

Here, σ denotes the Sigmoid function. It should be noted that in the IRN2Vec model, ${\text{R}}_{\text{x}}$ and ${\text{R}}_{\text{y}}$ are defined as the same matrix, which consists of the implicit feature vectors of all intersections in the road network, with each row corresponding to the latent representation of a road network node. During the training process, if nodes ${\text{v}}_{\text{x}}$ and ${\text{v}}_{\text{y}}$ satisfy the objective of a particular prediction task, their representations ${\text{R}}_{\text{x}}^{\prime }{\text{v}}_{\text{x}}$ and ${\text{R}}_{\text{y}}^{\prime }{\text{v}}_{\text{y}}$ in the latent space will move closer to each other; conversely, if they do not meet the objective, their representations will move away from each other in this space.

When training the parameters of the IRN2Vec model, multiple optimization objectives need to be set to accomplish the task, making the correct configuration of the objective function crucial. The IRN2Vec model is trained using a combination of backpropagation and asynchronous stochastic gradient ascent, a paradigm well-suited for distributed or edge-computing environments where training data or model updates can be processed across decentralized nodes. For a given training dataset, each sample is represented in the form of $\left\langle {\text{v}}_{\text{x}},{\text{v}}_{\text{y}},{\text{S}}_{\text{loc}}({\text{v}}_{\text{x}},{\text{v}}_{\text{y}}),{\text{S}}_{\text{tag}}({\text{v}}_{\text{x}},{\text{v}}_{\text{y}}),{\text{S}}_{\text{type}}({\text{v}}_{\text{x}},{\text{v}}_{\text{y}})\right\rangle$, and includes three Boolean features: ${\text{S}}_{\text{loc}}({\text{v}}_{\text{x}},{\text{v}}_{\text{y}})$ (indicating whether the intersection nodes vx and vy are within a specific K-meter range), ${\text{S}}_{\text{tag}}({\text{v}}_{\text{x}},{\text{v}}_{\text{y}})$ (reflecting whether they share the same intersection label), and ${\text{S}}_{\text{type}}({\text{v}}_{\text{x}},{\text{v}}_{\text{y}})$ (indicating whether they belong to the same intersection type). The IRN2Vec model is trained in parallel using a combination of backpropagation and asynchronous stochastic gradient ascent. During this process, the weights of ${\text{R}}_{\text{x}}$ and ${\text{R}}_{\text{y}}$ in each training sample are dynamically adjusted to maximize the objective function O. The objective function O consists of ${\text{O}}_{\text{loc}}({\text{v}}_{\text{x}},{\text{v}}_{\text{y}}),{\text{O}}_{\text{tag}}({\text{v}}_{\text{x}},{\text{v}}_{\text{y}}),{\text{O}}_{\text{type}}({\text{v}}_{\text{x}},{\text{v}}_{\text{y}})$, which are defined as follows.


$${\text{O}}_{\text{loc}}({\text{v}}_{\text{x}},{\text{v}}_{\text{y}})=\left\{ \;\;\;\begin{array}{c} {\text{P}}_{\text{loc}}({\text{v}}_{\text{x}},{\text{v}}_{\text{y}})\text{ if}{\text{ S}}_{\text{loc}}({\text{v}}_{\text{x}},{\text{v}}_{\text{y}})=\text{1}\;\\ \;\text{1}-{\text{P}}_{\text{loc}}({\text{v}}_{\text{x}},{\text{v}}_{\text{y}})\text{ if}{\text{ S}}_{\text{loc}}({\text{v}}_{\text{x}},{\text{v}}_{\text{y}})=\text{0} \end{array}\right.\;$$ (4)

$${\text{O}}_{\text{tag}}({\text{v}}_{\text{x}},{\text{v}}_{\text{y}})=\left\{ \;\;\;\begin{array}{c} {\text{P}}_{\text{tag}}({\text{v}}_{\text{x}},{\text{v}}_{\text{y}})\text{ if}{\text{ S}}_{\text{tag}}({\text{v}}_{\text{x}},{\text{v}}_{\text{y}})=\text{1}\;\\ \;\text{1}-{\text{P}}_{\text{tag}}({\text{v}}_{\text{x}},{\text{v}}_{\text{y}})\text{ if}{\text{ S}}_{\text{tag}}({\text{v}}_{\text{x}},{\text{v}}_{\text{y}})=\text{0} \end{array}\right.\;$$ (5)

$${\text{O}}_{\text{type}}({\text{v}}_{\text{x}},{\text{v}}_{\text{y}})=\left\{ \;\;\;\begin{array}{c} {\text{P}}_{\text{type}}({\text{v}}_{\text{x}},{\text{v}}_{\text{y}})\text{ if}{\text{ S}}_{\text{type}}({\text{v}}_{\text{x}},{\text{v}}_{\text{y}})=\text{1}\;\\ \;\text{1}-{\text{P}}_{\text{type}}({\text{v}}_{\text{x}},{\text{v}}_{\text{y}})\text{ if}{\text{ S}}_{\text{type}}({\text{v}}_{\text{x}},{\text{v}}_{\text{y}})=\text{0} \end{array}\right.\;$$ (6)

In Equations (4), (5), and (6), the purpose of ${\text{O}}_{\text{loc}}({\text{v}}_{\text{x}},{\text{v}}_{\text{y}}),{\text{O}}_{\text{tag}}({\text{v}}_{\text{x}},{\text{v}}_{\text{y}})$, and ${\text{O}}_{\text{type}}({\text{v}}_{\text{x}},{\text{v}}_{\text{y}})$ is to enable the IRN2Vec model to separately quantify the correct prediction of ${\text{S}}_{\text{loc}}({\text{v}}_{\text{x}},{\text{v}}_{\text{y}})$, ${\text{S}}_{\text{tag}}({\text{v}}_{\text{x}},{\text{v}}_{\text{y}})$, and ${\text{S}}_{\text{type}}({\text{v}}_{\text{x}},{\text{v}}_{\text{y}})$ for the training data. Specifically, for a given training sample, the objective of ${\text{O}}_{\text{loc}}({\text{v}}_{\text{x}},{\text{v}}_{\text{y}})$ is to maximize ${\text{P}}_{\text{loc}}({\text{v}}_{\text{x}},{\text{v}}_{\text{y}})$ when ${\text{S}}_{\text{loc}}({\text{v}}_{\text{x}},{\text{v}}_{\text{y}})=\text{1}$, and minimize it otherwise. The same logic applies to ${\text{O}}_{\text{tag}}({\text{v}}_{\text{x}},{\text{v}}_{\text{y}})$ and ${\text{O}}_{\text{type}}({\text{v}}_{\text{x}},{\text{v}}_{\text{y}})$.

During the optimization process, and in order to reduce computational complexity, we transform ${\text{O}}_{\text{loc}}({\text{v}}_{\text{x}},{\text{v}}_{\text{y}})$, ${\text{O}}_{\text{tag}}({\text{v}}_{\text{x}},{\text{v}}_{\text{y}})$, and ${\text{O}}_{\text{type}}({\text{v}}_{\text{x}},{\text{v}}_{\text{y}})$ into the maximization of $\text{log}{\text{O}}_{\text{loc}}({\text{v}}_{\text{x}},{\text{v}}_{\text{y}})$, $\text{log}{\text{O}}_{\text{tag}}({\text{v}}_{\text{x}},{\text{v}}_{\text{y}})$, and ${\text{logO}}_{\text{type}}({\text{v}}_{\text{x}},{\text{v}}_{\text{y}})$. These three objective functions are defined as follows.


$$\text{log}{\text{O}}_{\text{loc}}({\text{v}}_{\text{x}},{\text{v}}_{\text{y}})={\text{S}}_{\text{loc}}({\text{v}}_{\text{x}},{\text{v}}_{\text{y}})\text{log}{\text{P}}_{\text{loc}}({\text{v}}_{\text{x}},{\text{v}}_{\text{y}})+\left[\text{1}-{\text{S}}_{\text{loc}}({\text{v}}_{\text{x}},{\text{v}}_{\text{y}})\right]\text{log}\left[\text{1}-{\text{P}}_{\text{loc}}({\text{v}}_{\text{x}},{\text{v}}_{\text{y}})\right]$$ (7)

$$\text{log}{\text{O}}_{\text{tag}}({\text{v}}_{\text{x}},{\text{v}}_{\text{y}})={\text{S}}_{\text{tag}}({\text{v}}_{\text{x}},{\text{v}}_{\text{y}})\text{log}{\text{P}}_{\text{tag}}({\text{v}}_{\text{x}},{\text{v}}_{\text{y}})+\left[\text{1}-{\text{S}}_{\text{tag}}({\text{v}}_{\text{x}},{\text{v}}_{\text{y}})\right]\text{log}\left[\text{1}-{\text{P}}_{\text{tag}}({\text{v}}_{\text{x}},{\text{v}}_{\text{y}})\right]$$ (8)

$$\text{log}{\text{O}}_{\text{type}}({\text{v}}_{\text{x}},{\text{v}}_{\text{y}})={\text{S}}_{\text{type}}({\text{v}}_{\text{x}},{\text{v}}_{\text{y}})\text{log}{\text{P}}_{\text{type}}({\text{v}}_{\text{x}},{\text{v}}_{\text{y}})+\left[\text{1}-{\text{S}}_{\text{type}}({\text{v}}_{\text{x}},{\text{v}}_{\text{y}})\right]\text{log}\left[\text{1}-{\text{P}}_{\text{type}}({\text{v}}_{\text{x}},{\text{v}}_{\text{y}})\right]$$ (9)

Therefore, the overall objective function O can be defined as follows.


$$\text{O}=\alpha *{\text{L}}_{\text{loc}}+\beta *{\text{L}}_{\text{tag}}+(\text{1}-\alpha -\beta )*{\text{L}}_{\text{type}}$$ (10)

where L_* represent the corresponding log-likelihood losses, α and β are weighting coefficients.

During the first phase of constructing the IRN2Vec model, the LEIRN framework requires effective data sampling and training sample generation for road network nodes. The design of training data must address two key aspects: computational efficiency, by reducing the computational overhead associated with exhaustive enumeration through appropriate sampling methods, and sampling quality, ensuring that the training data comprehensively covers various types of intersections in the road network. Traditional network representation learning often relies on random walks for network structure sampling. However, this approach has limitations in the context of road networks, primarily because real-world user movement trajectories tend to exhibit directionality and purposefulness rather than being entirely random. To address this, the IRN2Vec model proposes using shortest paths as an alternative sampling strategy, which better aligns with the travel patterns of users in real-world road networks. In implementation, the Dijkstra algorithm, combined with a min-heap-optimized priority queue, is employed to randomly select intersection pairs and generate corresponding shortest path sequences. These sequences are then used to construct node sequences as training corpora for the model. Experimental results demonstrate that the shortest-path-based sampling method consistently outperforms the random walk strategy across multiple real-world tasks.

For each shortest path obtained through sampling, we extract the corresponding coordinate information (including longitude and latitude), intersection labels, and their associated types from the sequence of passed intersections, based on real annotation data. This processing aims to provide the necessary training data for subsequent research on geospatial local characteristics, correlations between intersections with identical labels, and relationships among intersections of the same category. It should be noted that in traditional graph representation learning methods, locality is typically characterized by hop-based neighborhood relationships.

In the IRN2Vec model, for any two road network nodes ${\text{v}}_{\text{x}}$ and ${\text{v}}_{\text{y}}$ within the same window, the model generates a positive sample representing their geospatial proximity. Additionally, the model analyzes whether the node pair shares identical intersection labels and types to enrich the semantic features of the samples. Beyond positive samples, the model also employs a negative sampling strategy for contrastive learning. Drawing on the negative sampling method of Word2Vec [14], it constructs corresponding negative samples for model training by randomly replacing one of the nodes in each positive sample (either ${\tilde{\text{v}}}_{\text{x}}$ or ${\tilde{\text{v}}}_{\text{y}}$) with another arbitrary intersection node.

Computational Complexity Analysis: The following analysis evaluates the scalability of the IRN2Vec learning algorithm in terms of time and space complexity. It focuses on the model’s efficiency in processing graph data, independent of the underlying data storage architecture. The complexity of the IRN2Vec framework stems from two stages. (1) Data Generation: Using a min-heap optimized Dijkstra algorithm, the complexity for sampling one shortest path from a random OD pair is O(|E| + |V|\log|V|), where |V| and |E| are the number of nodes and edges. This is a pre-processing step performed once. (2) Model Training: Each training step involves a forward/backward pass for a node pair, which is O(d) where d is the embedding dimension, independent of graph size. The overall training complexity is GATar in the number of generated samples and dd. Comparatively, GCN’s random walk generation is O(L ⋅ N ⋅ kˉ) where L is walk length, N is the number of walks, and kˉ is average node degree, and its training (using Skip-gram) is similar in complexity to ours per sample. GAT explicitly computes node-pair similarities, leading to a complexity related to |E|. While our shortest-path sampling has a higher per-sample generation cost than a random walk step, it yields more informative samples, often requiring fewer total samples for convergence.

### Comparative experiments

This subsection provides a concise table listing the key hyperparameters for all models, including our IRN2Vec and the baseGATs (UID, GCN, GAT).

Example Table Row for GCN: Embedding dimension d = 128, number of walks per node r = 10, walk length l = 80, window size w = 10, negative samples k = 5.

For GAT: We specify the order (GAT(1st), GAT(2nd), or combined) and the corresponding negative sampling parameters used.

Statement: We clarify that for all embedding-based baseGATs, we performed a grid search over these key parameters and report the results from their optimal configurations to ensure a fair comparison.

The OpenStreetMap (OSM) road network graphs for San Francisco, Porto, and Tokyo contain 395,290 intersections and 536,746 road segments in total. For the traffic signal classification task, the experiments utilized 4,521, 7,842, and 12,856 intersections from the three cities, respectively. For the pedestrian crossing classification task, the experiments utilized 5,021, 6,937, and 14,367 intersections. In all cases, the data was split into a 90% training set and a 10% test set.

Intersection labels (traffic signals, pedestrian crossings) were extracted from the highway and crossing tags in OSM. While OSM provides extensive coverage, its annotation is community-driven and may contain inconsistencies (e.g., missing or outdated tags). To ensure a reliable ground truth for evaluation, we performed a two-step verification: (1) Automated filtering to remove nodes with conflicting tags, and (2) Manual spot-checking of a random 5% sample for each city and label type. This process confirmed an estimated annotation accuracy exceeding 95% for the retained samples, which we deem sufficient for robust model training and evaluation.

To evaluate the effectiveness of the IRN2Vec model, this study employs two real-world datasets for assessment: a road network dataset [12] and a trajectory dataset [15]. The road network dataset provides intersection and road segment data, while the trajectory dataset offers movement paths and corresponding travel times. Furthermore, comparative experiments are conducted between the IRN2Vec model and existing network representation learning models to comprehensively assess its performance.

Although this study uses anonymized, publicly available trajectory datasets, we acknowledge the broader ethical and privacy concerns associated with GPS mobility data. We advocate for and adhere to the principles of data minimization, using only data necessary for research purposes, and support the development of privacy-preserving techniques like federated learning or differential privacy for future real-world deployments.

The road network dataset comprises three distinct urban road systems—San Francisco, Porto, and Tokyo—containing 395,290 intersections, 536,746 road segments, and 47,925 intersection labels; the trajectory dataset includes 153,765,335 GPS points and 1,950,218 trajectories, with an average time interval of 14.74 seconds. It is important to note that all personally identifiable information (PII) has been rigorously anonymized and removed from the publicly available trajectory data used in this study. To characterize the sampled data, the average shortest-path length (in number of intersection hops) for the randomly selected OD pairs was 12.3 for San Francisco, 18.7 for Porto, and 22.1 for Tokyo, reflecting the differing scales and densities of the urban networks.

The use of real-world spatiotemporal data, even when anonymized, necessitates a discussion of privacy. Our framework employs a privacy-by-design principle at the data ingestion stage. We rely on pre-processed, aggregated trajectory data from which all direct identifiers have been stripped. Furthermore, our shortest-path sampling strategy inherently contributes to privacy preservation. By generating synthetic path sequences based on network topology rather than relying solely on raw individual trajectories, it abstracts away from specific user travel patterns, reducing the risk of re-identification. However, we acknowledge that advanced inference attacks remain a concern in ITS data analytics. Future work will explore integrating formal privacy guarantees, such as differential privacy, into the representation learning process itself to further mitigate potential risks.

While the available trajectory dataset provides rich empirical movement patterns, we opted for a shortest-path sampling strategy for two primary reasons: (1) Generalization & Coverage: Real trajectories are often sparse and concentrated on popular routes, potentially under-representing large portions of the network. Shortest-path sampling ensures comprehensive coverage of all possible node pairs and paths, providing a more complete structural context for every intersection. (2) Efficiency & Stability: Generating a massive number of synthetic shortest paths on-demand is computationally straightforward and consistent. In contrast, processing and sampling from millions of raw, noisy GPS trajectories for each training epoch is significantly more resource-intensive and can introduce instability due to varying data density and quality.

**Fig 3:**
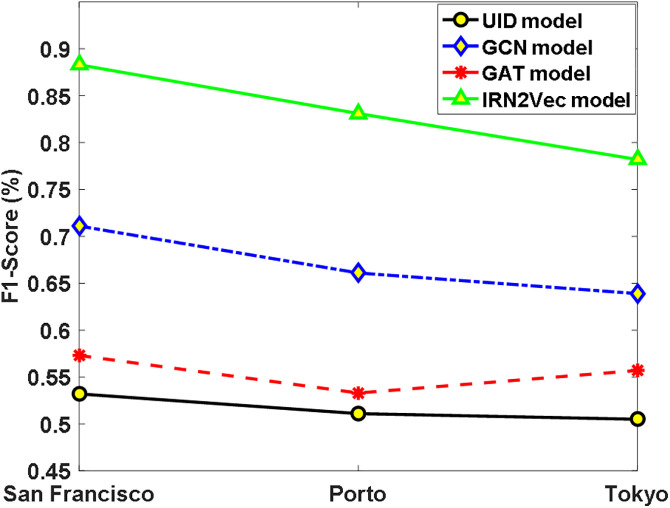
Traffic Signal Classification Results Across the Three Cities.

**Fig 4:**
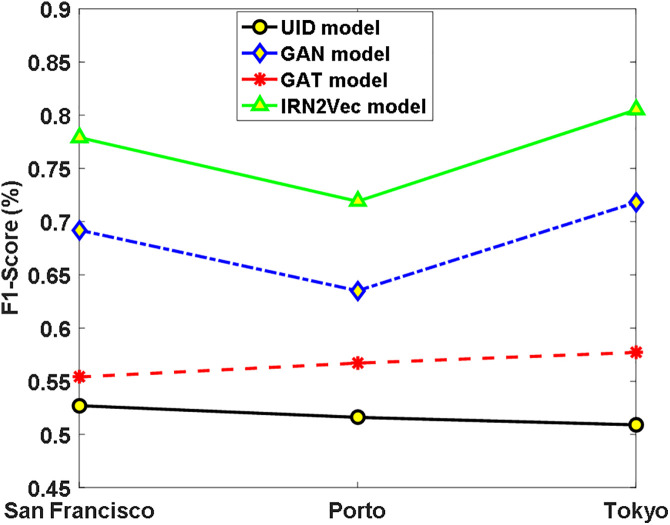
Categorization Results of Pedestrian Crossings in Three Cities.

To objectively evaluate the performance of the IRN2Vec model in node classification, a binary classification task was employed to assess the model. This task includes traffic signal classification and crosswalk intersection classification, aimed at determining whether a road network intersection contains traffic signal signs or crosswalk signs. We have updated the description of evaluation metrics from solely F1-Score to include Precision, Recall, and Accuracy alongside F1-Score. The reported results in the text and figures (Figs 3–4) have been updated accordingly.

First, a traffic signal classification experiment was conducted. The intersection data from the road network dataset was split into two parts: 90% for the training set and the remaining 10% for the test set. The training set was used to train an SVM classifier, and the model’s performance was evaluated using the test set. Existing network representation learning models, namely UID [11], GCN [16], and GAT [17], were compared with the proposed IRN2Vec model in the experiment. The F1-Score [18] was adopted as the evaluation metric, and the experimental results are shown in Fig 3.

According to Fig 3, all representation learning-based models outperform the UID model, demonstrating the effectiveness of incorporating road network intersection representations in traffic signal classification. Furthermore, the IRN2Vec model achieves an average F1-Score that is 31.6%, 16.2%, and 27.8% higher than that of the UID, GCN, and GAT models, respectively, significantly surpassing the performance of the other three models. These results also validate the argument presented in this paper that the shortest path-based sampling approach is clearly superior to random walk-based sampling methods such as GCN.

To demonstrate the robustness of the IRN2Vec model, a pedestrian crossing intersection classification experiment was subsequently conducted. This task aims to infer whether an intersection in the road network has a pedestrian crossing sign. The experimental setup remained consistent with that of the traffic signal classification task. The experimental samples were selected from the road network datasets, comprising 5,021 intersections from San Francisco, 6,937 from Porto, and 14,367 from Tokyo. For each city, 90% of the intersection data was used as the training set, while the remaining 10% served as the test set. The experimental results are shown in Fig 4.

As shown in Fig 4, all representation learning-based models outperform the UID model, demonstrating the effectiveness of utilizing road network intersection representation learning for pedestrian crossing classification. Furthermore, the IRN2Vec model achieves an average F1-Score that is 25.1%, 8.6%, and 20.2% higher than that of the UID, GCN, and GAT models, respectively, indicating its superior performance and robustness compared to the other three models.

To validate the applicability of the road network intersection embeddings generated by the IRN2Vec model in general intelligent transportation tasks, this study adopts travel time prediction as a specific application scenario, and systematically designs the experimental procedures for travel time prediction, the workflow for road network mapping, and the comparative experiments between the IRN2Vec model and other baseGAT models.

Travel time prediction, as a regression task, aims to estimate the travel duration for a given moving path within a road network; the experiment utilizes publicly available trajectory datasets from San Francisco, Porto, and Tokyo to construct training and testing data, but to ensure a valid evaluation, the arrival time information of raw GPS points in the trajectories is deliberately masked during testing to prevent models from making predictions by simply accumulating fixed intervals between sampling points. To achieve this, the study first employs a Hidden Markov Model-based map matching technique [19] to project raw GPS trajectories onto the underlying road network, thereby converting them into sequences of consecutive intersection nodes, ultimately defining the experimental objective as accurately predicting the total travel time based on this sequence of intersections.

**Fig 5:**
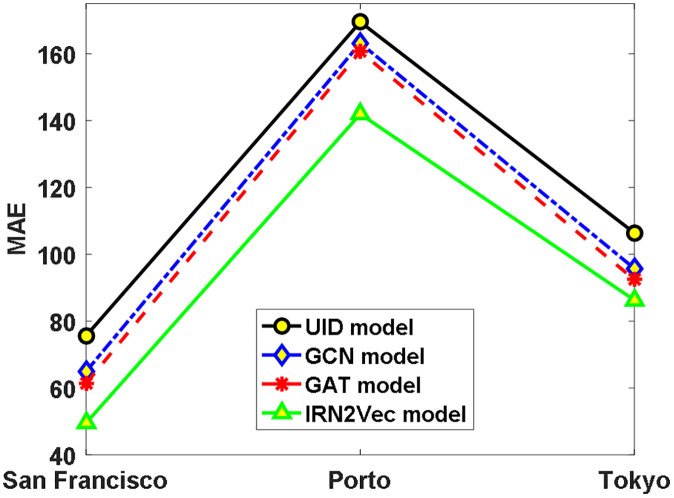
Comparative Results of Travel Time Prediction in Three Cities.

To accomplish the travel time prediction task, a regression model was constructed in the experiments, which centers around a Long Short-Term Memory (LSTM) recurrent network [20] followed by three fully connected layers with dimensions of 128, 128, and 1, respectively. The model takes as input a movement path represented by a sequence of intersection embedding vectors, processes each intersection state step by step through the LSTM, and ultimately outputs the predicted total travel time. The model’s performance was evaluated using the Mean Absolute Error (MAE) [21], defined as the average of the absolute differences between the predicted values and the ground truth travel times (in seconds) extracted from raw trajectory data. The experimental setup remained consistent with that of prior traffic signal classification experiments. The results are presented in (Fig 5).

As observed from Fig 5, all embedding-based model methods outperform the UID model, indicating that using implicit feature vectors of road network intersections for travel time prediction is effective. Furthermore, the Mean Absolute Error (MAE) of the IRN2Vec model is reduced by 24.6%, 15.4%, and 12.2% compared to the UID, GCN, and GAT models, respectively. This demonstrates that the IRN2Vec model achieves smaller errors and superior performance over the other three models, reaffirming that capturing the movement behavior of mobile road users through shortest-path sampling can significantly enhance the efficiency of road network-based applications.

Runtime Comparison: We report the average total runtime (including data preprocessing/sampling and model training) for each model on the San Francisco dataset on a single GPU (NVIDIA RTX 3090). UID, as a non-neural heuristic, was the fastest (<1 min). GCN required ~25 minutes, GAT ~ 42 minutes, and IRN2Vec ~ 55 minutes. While IRN2Vec has a higher upfront cost due to shortest-path computation, its training converges efficiently. The performance gains (e.g., 16.2–31.6% higher F1 than baseGATs) justify this moderate increase in computational time, which remains feasible for large-scale applications.

To decouple the performance gains attributed to the shortest-path sampling strategy from those due to the injection of geospatial and semantic side-information (labels and types), we conducted an ablation study on the San Francisco dataset. We compared four variants of our framework:

1. SP-Multi: The full IRN2Vec model using Shortest-Path sampling and Multi-task learning (all three objectives: Loc, Tag, Type).
2. SP-Struct: Uses Shortest-Path sampling but only the geographical proximity objective (Loc), effectively making it a structure-only model that learns from spatial neighborhoods.
3. RW-Multi: Uses Random Walk sampling (like GCN) but with our Multi-task learning objectives (Loc, Tag, Type).
4. RW-Struct: Uses Random Walk sampling and only the geographical proximity objective. This is most similar to classic network embedding methods like GCN but defined over spatial neighborhoods.

The F1-Scores for the traffic signal classification task are analyzed. The results show that:

Comparing SP-Struct vs. RW-Struct: Using shortest-path sampling alone (without side-information) yields a 7.3%

improvement in F1-Score. This confirms that the proposed sampling strategy better captures meaningful topological and behavioral patterns than random walks, independent of additional node attributes.

Comparing SP-Multi vs. SP-Struct: Adding the tag and type objectives (side-information) to the shortest-path sampler further improves performance by 9.1%. This validates the effectiveness of the multi-task learning framework in integrating semantic homogeneity.

Comparing SP-Multi (our full model) vs. RW-Multi: The combination of shortest-path sampling and multi-task learning achieves the best performance, surpassing the random-walk-based multi-task model by 10.8%. This demonstrates that both components—behaviorally-realistic sampling and multi-source feature integration—are complementary and essential for the superior performance of IRN2Vec.“

Sensitivity Analysis of Weighting Coefficients: Performance is suboptimal if one objective dominates.The model is robust across a wide range of values (α∈[0.2,0.5], β∈[0.2,0.4]). The best performance (F1-Score = 0.873 on San Francisco data) was achieved at α = 0.3,β = 0.3, a balanced configuration used in all main experiments.

Limitations and Future Work:

(a) Label Dependence: The model’s performance for tasks like signal classification depends on the availability and quality of semantic labels (e.g., from OSM). Its applicability may be limited in regions with sparse or inconsistent annotations. Future work could explore weakly-supervised or self-supervised paradigms to mitigate this dependency.
(b) Computational Scalability: While efficient for city-scale networks, the pairwise relationship modeling and shortest-path sampling may face challenges on continental-scale networks with millions of nodes. Investigating more scalable sampling strategies (e.g., hierarchical) and leveraging graph partitioning techniques are important directions for scaling.
(c) Sensitivity to Noisy Data: The model assumes trajectory data is available for shortest-path sampling. Its performance could degrade with extremely sparse or noisy GPS data, which may fail to capture true travel patterns. Incorporating data imputation or uncertainty-aware learning could enhance robustness.
(d) Transferability Across Cities: While we demonstrate cross-city experiments, the model’s transferability to regions with vastly different network typologies (e.g., grid vs. organic) requires further validation. Developing topology-agnostic or meta-learning-based adaptation mechanisms is a promising avenue.

## Conclusions

This study employs the LEIRN framework and the IRN2Vec model to conduct representational learning on road network intersections, exploring their geographical locality and mobility behavior characteristics to construct intersection embedding vectors for supporting intelligent transportation applications. Through experiments and analysis on large-scale real-world datasets, the following conclusions are drawn:

(1) A two-stage LEIRN framework is proposed, which effectively captures diverse geospatial characteristics between road intersections. In the first stage, a shortest-path-based sampling method is employed to construct training data for the IRN2Vec model; in the second stage, multi-objective learning integrates features including geographical locality, label consistency, and customized types to generate highly expressive intersection embedding representations.
(2) The proposed IRN2Vec model demonstrates the capability to automatically learn implicit feature vectors of road intersections, thereby effectively supporting various downstream intelligent transportation tasks including traffic flow prediction, route planning, and intersection type recognition. Experimental results indicate that the model can adequately capture the intrinsic structural and semantic information of road networks.
(3) To validate the model performance, comparative experiments were conducted between IRN2Vec and several mainstream representation learning models. The results demonstrate that the proposed method outperforms other prevalent models across multiple evaluation metrics, exhibiting superior representation quality, robustness, and generalization capability while adapting to road network data of varying scales and structures.
(4) The primary contribution of this research lies in proposing a multi-source feature-integrated intersection representation learning framework capable of automatically extracting discriminative intersection embeddings from raw trajectory data, thereby providing foundational feature support for intelligent transportation systems. The research outcomes can be extensively applied to scenarios including traffic state perception, travel behavior analysis, and dynamic road network optimization, consequently facilitating the advancement of precise and intelligent management in smart city transportation systems. The primary contribution of this research lies in proposing a multi-source feature-integrated intersection representation learning framework. This framework utilizes data from the IoT infrastructure of smart cities to automatically extract discriminative intersection embeddings, thereby providing foundational feature support for intelligent transportation systems.
(5) The model’s training paradigm and efficient inference align with emerging Edge Computing architectures for ITS. The asynchronous training and the lightweight final embedding lookup table (requiring only O(d) memory per intersection) make IRN2Vec amenable to deployment in edge networks. Once trained, the model and its static embeddings can be securely distributed and managed. For dynamic scenarios where embeddings are updated, integrating with blockchain-based IoT platforms could provide a robust mechanism for ensuring the integrity and auditability of embedding versions during transmission and storage across distributed edge nodes, preventing tampering and ensuring consistency—a valuable direction for future secure system integration.
(6) The LEIRN framework’s design supports effective integration into scalable ITS architectures. The framework operates in two distinct phases: an offGAT training phase and an onGAT inference phase. The training phase, while computationally intensive, is a one-time or periodic batch process. Our complexity analysis shows its scalability for large networks. More critically for real-time response, the inference phase is extremely lightweight—generating a prediction involves only a fast embedding lookup and a simple dot product. This makes the trained IRN2Vec model ideal for deployment within distributed edge computing architectures. Edge nodes can store the compact embedding matrix locally. During data surges, this design minimizes latency and bandwidth needs, as real-time analysis requires only local vector operations without constant cloud queries. Thus, the framework effectively handles real-time demands by shifting computational load to offGAT training and enabling low-latency, distributed inference at the edge, which inherently buffers against cloud-side data surges.

Finally, while this work demonstrates the scalability of the learning algorithm on large-scale network data, we note that integrating the model with resilient data infrastructure—such as cloud platforms for distributed training or blockchain for secure, auditable data provenance in multi-stakeholder environments—remains a valuable direction for future system-level integration and deployment.

## Supporting information

S1 File Lab Data. (DOCX)
