# Supplementary material for: IRN2Vec: A representation learning model for road network intersections by integrating geospatial attributes and travel behaviors
Source: PLoS One. 2026 Mar 13;21(3):e0344448. doi: 10.1371/journal.pone.0344448 (PMC12987413; doi:10.1371/journal.pone.0344448)
Supplement: S1 File — (DOCX) [file pone.0344448.s001.docx]

Supporting Information: Lab data

**Comparison data of F1 scores of traffic signal classification**

| model |  | | |
| --- | --- | --- | --- |
| UID | Test step number | | |
|  | 1 | 2 | 3 |
|  | F1 score | | |
|  | 0.532 | 0.511 | 0.505 |
|  | Average F1-Score | | |
|  | 0.516 | | |
| DeepWalk | Test step number | | |
|  | 1 | 2 | 3 |
|  | F1 score | | |
|  | 0.711 | 0.661 | 0.639 |
|  | Average F1-Score | | |
|  | 0.670 | | |
| LINE | Test step number | | |
|  | 1 | 2 | 3 |
|  | F1 score | | |
|  | 0.573 | 0.533 | 0.557 |
|  | Average F1-Score | | |
|  | 0.554 | | |
| This article's method  (IRN2Vec) | Test step number | | |
|  | 1 | 2 | 3 |
|  | F1 score | | |
|  | 0.883 | 0.831 | 0.782 |
|  | Average F1-Score | | |
|  | 0.832 | | |

**Comparison data of F1 scores of pedestrian crossings categorization**

| model |  | | |
| --- | --- | --- | --- |
| UID | Test step number | | |
|  | 1 | 2 | 3 |
|  | F1 score | | |
|  | 0.527 | 0.516 | 0.509 |
|  | Average F1-Score | | |
|  | 0.517 | | |
| DeepWalk | Test step number | | |
|  | 1 | 2 | 3 |
|  | F1 score | | |
|  | 0.692 | 0.635 | 0.718 |
|  | Average F1-Score | | |
|  | 0.682 | | |
| LINE | Test step number | | |
|  | 1 | 2 | 3 |
|  | F1 score | | |
|  | 0.554 | 0.567 | 0.577 |
|  | Average F1-Score | | |
|  | 0.566 | | |
| This article's method  (IRN2Vec) | Test step number | | |
|  | 1 | 2 | 3 |
|  | F1 score | | |
|  | 0.779 | 0.719 | 0.805 |
|  | Average F1-Score | | |
|  | 0.768 | | |

**Comparison data of MAE of Travel Time Prediction**

| model |  | | |
| --- | --- | --- | --- |
| UID | Test step number | | |
|  | 1 | 2 | 3 |
|  | MAE | | |
|  | 75.62 | 169.49 | 106.39 |
|  | Average MAE | | |
|  | 117.16 | | |
| DeepWalk | Test step number | | |
|  | 1 | 2 | 3 |
|  | MAE | | |
|  | 64.97 | 163.17 | 95.74 |
|  | Average MAE | | |
|  | 107.96 | | |
| LINE | Test step number | | |
|  | 1 | 2 | 3 |
|  | MAE | | |
|  | 61.37 | 160.65 | 92.43 |
|  | Average MAE | | |
|  | 104.82 | | |
| This article's method  (IRN2Vec) | Test step number | | |
|  | 1 | 2 | 3 |
|  | MAE | | |
|  | 49.54 | 141.96 | 86.29 |
|  | Average MAE | | |
|  | 92.59 | | |
